# Quantum dot labeling of mesenchymal stem cells

**DOI:** 10.1186/1477-3155-5-9

**Published:** 2007-11-07

**Authors:** Barbara J Muller-Borer, Maria C Collins, Philip R Gunst, Wayne E Cascio, Alan P Kypson

**Affiliations:** 1 Department of Internal Medicine, The Brody School of Medicine at East Carolina University, Greenville, NC-27834, USA; 2 Department of Surgery, The Brody School of Medicine at East Carolina University, Greenville, NC-27834, USA

## Abstract

**Background:**

Mesenchymal stem cells (MSCs) are multipotent cells with the potential to differentiate into bone, cartilage, fat and muscle cells and are being investigated for their utility in cell-based transplantation therapy. Yet, adequate methods to track transplanted MSCs *in vivo *are limited, precluding functional studies. Quantum Dots (QDs) offer an alternative to organic dyes and fluorescent proteins to label and track cells *in vitro *and *in vivo*. These nanoparticles are resistant to chemical and metabolic degradation, demonstrating long term photostability. Here, we investigate the cytotoxic effects of *in vitro *QD labeling on MSC proliferation and differentiation and use as a cell label in a cardiomyocyte co-culture.

**Results:**

A dose-response to QDs in rat bone marrow MSCs was assessed in Control (no-QDs), Low concentration (LC, 5 nmol/L) and High concentration (HC, 20 nmol/L) groups. QD yield and retention, MSC survival, proinflammatory cytokines, proliferation and DNA damage were evaluated in MSCs, 24 -120 hrs post QD labeling. In addition, functional integration of QD labeled MSCs in an *in vitro *cardiomyocyte co-culture was assessed. A dose-dependent effect was measured with increased yield in HC vs. LC labeled MSCs (93 ± 3% vs. 50% ± 15%, p < 0.05), with a larger number of QD aggregates per cell in HC vs. LC MSCs at each time point (p < 0.05). At 24 hrs >90% of QD labeled cells were viable in all groups, however, at 120 hrs increased apoptosis was measured in HC vs. Control MSCs (7.2% ± 2.7% vs. 0.5% ± 0.4%, p < 0.05). MCP-1 and IL-6 levels doubled in HC MSCs when measured 24 hrs after QD labeling. No change in MSC proliferation or DNA damage was observed in QD labeled MSCs at 24, 72 and 120 hrs post labeling. Finally, in a cardiomyocyte co-culture QD labeled MSCs were easy to locate and formed functional cell-to-cell couplings, assessed by dye diffusion.

**Conclusion:**

Fluorescent QDs label MSC effectively in an *in vitro *co-culture model. QDs are easy to use, show a high yield and survival rate with minimal cytotoxic effects. Dose-dependent effects suggest limiting MSC QD exposure.

## Background

Cell transplantation therapy using adult derived bone marrow mesenchymal stem cells (MSCs) is currently being investigated as a potential therapy to treat injured heart tissue [1,2]. Transplanted MSCs are expected to engraft, differentiate and remodel in response to the surrounding cardiac microenvironment resulting in tissue regeneration and functional repair. The mechanisms underlying MSC engraftment and electrical and mechanical integration with host cardiac tissue are not understood. In part, this is due to limited methods to track MSCs *in vivo*, precluding long-term functional studies of transplanted cells. Current methods for labeling MSCs include ultra small iron particles (superparamagnetic iron oxide) [3], radioactive labels ([^111^In] indium oxine) [4], and organic fluorescent dyes loaded exogenously into cells [5] or fluorescent proteins expressed by the cells [6]. Yet, chemical and metabolic degradation, reduced photo-stability and signal quality [7] compromise *in vitro *and *in vivo *cell labeling and tracking.

Nanotechnology is focused on the development of nanoscale materials and devices with use in biomedicine for drug delivery, diagnostics, imaging and cell tracking. Quantum dots (QDs) are fluorescent semiconductor nanoparticles, recently adopted for use in *in vitro *and *in vivo *bioimaging [8,9]. Reported advantages of QDs include a narrow band emission and broadband excitation with a high quantum yield, photostability, luminescence and resistance to chemical and metabolic degradation [8,10,11]. These properties make QDs amenable to multicolor imaging applications and the tracking of live cells [12]. Reports in the literature suggest that QDs are non-cytotoxic [8,13], while recent data suggests QD cytotoxicity due to different physicochemical properties, dose and exposure concentrations [14-18]. Most QD applications have utilized non-mammalian or cancer cells with only a few studies examining deleterious effects of QDs in MSCs [8,18-20].

In the present study, rat bone marrow MSCs were used to evaluate QD exposure on labeled MSC yield, QD retention and proliferation. In addition, proinflammatory cytokines and DNA damage were examined to measure cellular responses to QD stimuli *in vitro*. We assessed the ability to track QD labeled MSCs in an *in vitro *cardiomyocyte co-culture. Finally, using a dye transfer assay functional cell-to-cell coupling of the MSCs with cardiomyocytes was assessed. Our results show bright, photostable QD labeled MSCs coupled functionally with cardiomyocytes in co-culture, indicating that QDs show promise as a cell labeling agent for studies tracking the fate of MSCs in culture. Dose-dependent cytotoxic effects suggest that QD exposure be limited to low concentrations for long-term *in vivo *cell transplantation studies.

## Results

### QD yield and intracellular distribution

**Figure 1:**
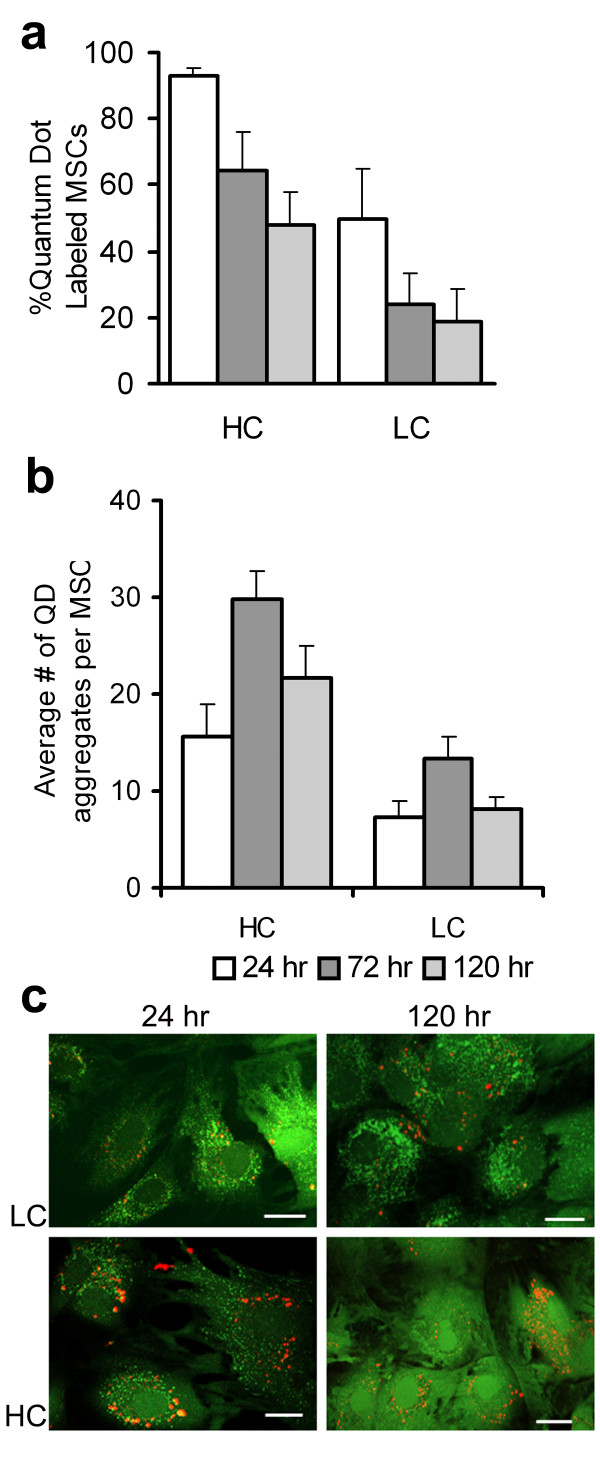
**Intracellular QD yield, retention and distribution in expanding MSC cultures**. **a**. Flow cytometry results of QD positive MSCs in HC and LC groups at 24, 72 and 120 hrs post labeling. A dose-dependent effect is shown with increased HC vs. LC QD labeled MSCs detected at each time point (p < 0.05). **b**. Quantitative imaging results show a greater number of QD aggregates in the HC vs. LC MSCs at each time point (p < 0.05). The average number of intracellular QD aggregates increased from 24 to 72 hrs in both groups of MSCs (p < 0.05). No statistically significant changes in QD aggregates were measured from 72 – 120 hr. **c**. Representative confocal fluorescent images of LC and HC MSCs co-labeled with calcein (green) at 24 and 120 hrs. Each image represents a 1 μm thick optical slice establishing a peri-nuclear intracellular distribution of QDs. As the MSCs proliferated QDs remained bright and easy to detect. Scale bar 20 μm.

**Figure 2:**
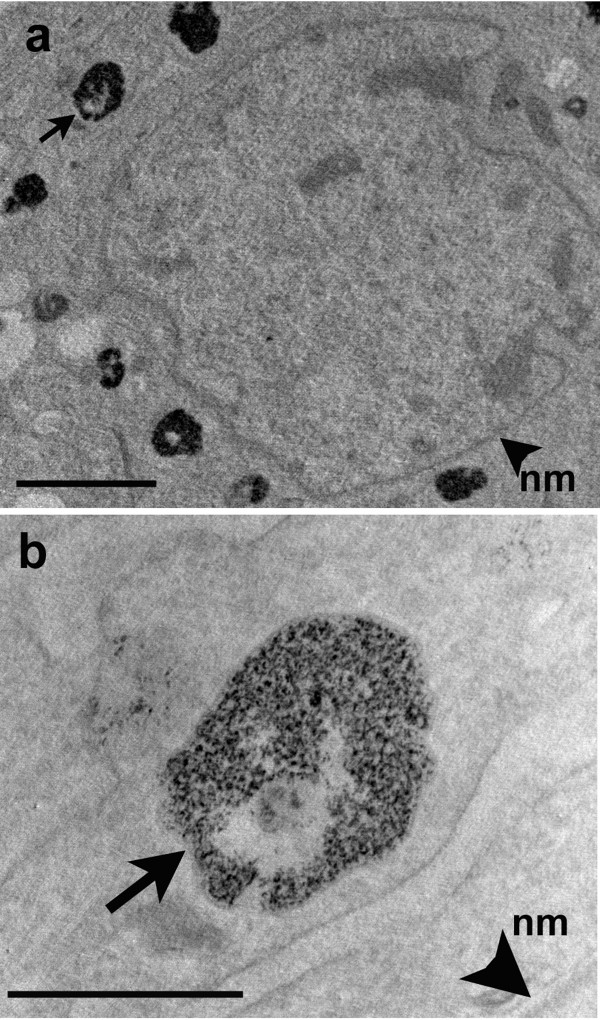
**TEM of QD labeled MSC**. **a**. Low magnification, representative image of MSC with QD nanocrystal aggregates in endosomal vesicles around nuclear membrane (nm, arrowhead). Scale bar 2 μm. **b**. High magnification of enlarged single vesicle (arrow, a and b) showing individual QDs. Scale bar 500 nm.

Flow cytometry and confocal microscopy assessed intracellular QD labeling at 24, 72 and 120 hrs in Control (media only), High QD concentration (HC, 20 nmol/L) and Low QD concentration (LC, 5 nmol/L) MSC groups. Flow cytometry results, shown in Figure 1a, illustrate a dose-dependent effect with increased HC vs. LC QD labeled MSCs (93% ± 3% vs. 50% ± 15%, p < 0.05) measured 24 hrs post QD labeling. As the MSCs proliferated in culture the number of QD labeled MSCs detected with flow cytometry decreased to 64% ± 12% vs. 25% ± 9% at 72 hrs and 48% ± 10% vs. 19% ± 10% at 120 hrs in the HC vs. LC MSCs (p < 0.05). Confocal images were used to quantitate intracellular QD aggregates. For each group (HC and LC) and at each time point (24, 72, 120 hr) an average of 100 cells were evaluated. The average number of QD aggregates in the HC MSCs was greater than in the LC MSCs at each time point (p < 0.05), shown in Figure 1b. Similar to findings by Seleverstov et. al. [18] and Rosen et. al. [20] QDs tended to form large intracellular aggregates in the MSCs. This observation resulted in the average number of QD aggregates recorded in MSCs increasing from 24 to 72 hrs in both groups of MSCs (p < 0.05). No statistically significant differences in intracellular QD aggregate numbers were observed from 72 to120 hrs. Figure 1c illustrates QD location and distribution in live MSCs at 24 and 120 hrs post labeling. For both exposure groups QDs were detected with confocal fluorescence microscopy and distributed in the cytosol with no QDs detected in the nucleus. TEM images of MSCs labeled with QDs are shown in Figure 2. QD aggregates were found in the MSC vesicles (panel a) around the nucleus (similar to the confocal images) in agreement with Seleverstov et. al. [18]. At high resolution (panel b, 10^5 ^× magnification) individual QDs were observed in the vesicles with an average diameter of 9.8 ± 1.0 nm.

### MSC survival

**Figure 3:**
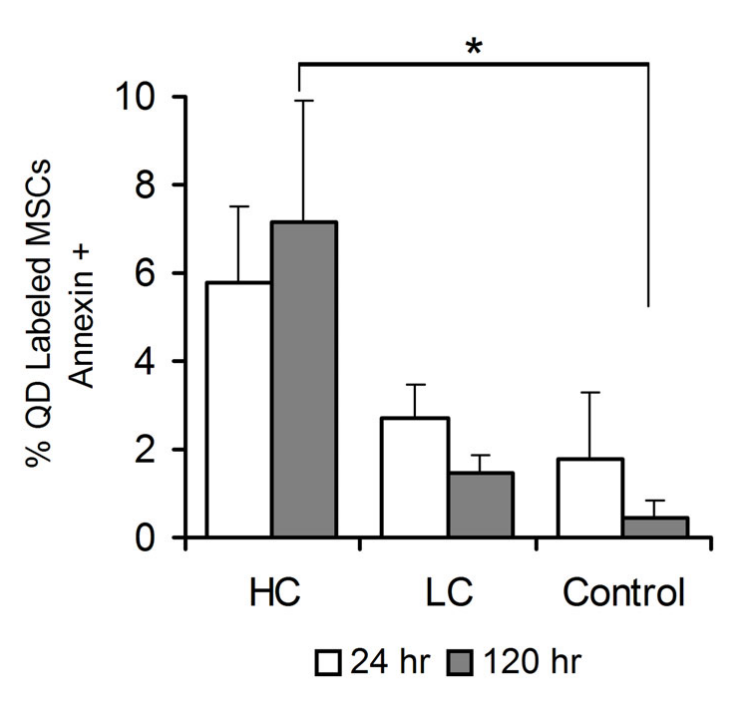
**Effect of QD labeling on apoptotic cell death in Control and QD labeled MSCs**. Flow cytometry results at 24, 72 and 120 hrs after QD labeling. The percent of annexin positive QD labeled MSCs was similar for HC, LC and Control MSCs at 24 hrs post labeling. Increased apoptosis was observed in HC vs. Control MSCs at 120 hrs (*p < 0.05). No difference in apoptosis was detected in LC vs. Control MSCs.

To determine whether QDs induced apoptotic cell death, MSCs were labeled with Annexin V and flow cytometry analysis assessed MSC viability identifying apoptosis in the cell population post QD labeling. Shown in Figure 3, at 24 hrs > 90% of QD labeled MSCs were viable in LC, HC and Control MSCs (no QD exposure). Apoptosis increased in HC MSCs vs. Control MSCs 120 hr post QD labeling (7.2% ± 2.7% vs. 0.5% ± 0.4%, p < 0.05).

### Cytokine release

Monocyte Chemoattractant Protein-1 (MCP-1), Interleukin-6 (IL-6), Interleukin-1 Beta (IL-1β) and Tumor Necrosis Factor alpha (TNF-α) levels were measured 24 hrs post QD labeling. The levels of MCP-1 and IL-6 doubled in HC MSCs compared to the LC MSCs and Control MSCs. There was no difference in the levels of MCP-1 and IL-6 in LC vs. Control MSCs. No dose response (HC vs. LC) or increase above Control MSCs was measured in cytokine levels of IL-1β and TNF-α 24 hrs post QD labeling.

### Cell proliferation and DNA damage

No change in MSC metabolic activity as a measure of proliferation was recorded at 24, 72, and 120 hrs after QD labeling in LC, HC and Control MSCs. DNA damage was assessed with the micro-scale cell-based comet bioassay. Single and double strand DNA damage was identified by increased dispersion patterns of the comet tail and reported as tail moment. The results, (data not shown) suggest a trend toward increased DNA damage with increased QD dose. Nevertheless, there was no statistically significant difference in comet tail moment measured in LC and HC MSCs compared to Control MSCs.

### In vitro model

**Figure 4:**
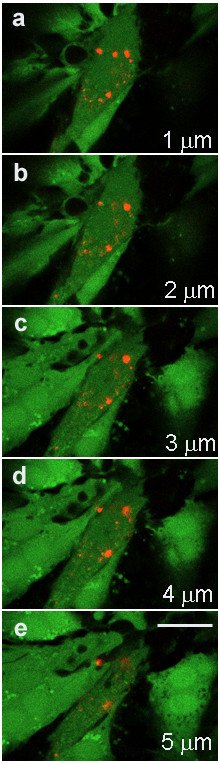
**QD labeled MSC in cardiac myocyte co-culture at 7 days**. Images show optical sections acquired as a confocal Z stack with 1-μm spacing. Image **a **shows QD labeled MSC above cardiac myocytes. As images advance into the cell culture (a – e, towards coverslip) the QD labeled MSC is shown adjacent to and surrounded by cardiac myocytes. All cells were labeled with the cytosolic fluoroprobe calcein AM. QDs are preferentially localized in the MSC. No QDs were found to be localized in the cardiac myocytes. All images were acquired with an oil immersion 40× objective. Scale bar 20 μm.

**Figure 5:**
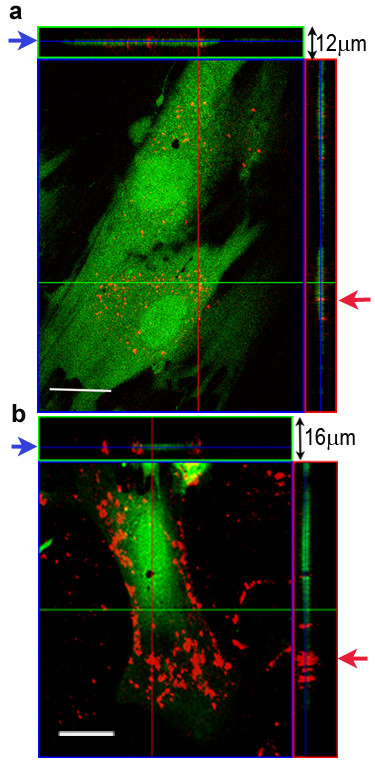
**3D distribution and localization of QDs in MSCs and cardiac myocytes**. A cut view through 12 (a) and16 (b) superimposed optical sections illustrating the 3D distribution of QDs in MSC and Cardiac Myocyte cultures 24 hrs post QD labeling. The sections shown are taken from the intracellular space of the cells indicated by the blue arrows with the red (vertical) and green (horizontal) crosshairs aligned near QD aggregates. **a**. The QDs are homogeneously distributed through the MSC cytosol, have not formed large aggregates and are clearly visualized in the intracellular space as indicated by the red arrow. Scale bar = 20 μm. **b**. 3D distribution of QDs in the cardiomyocyte culture clearly show QDs located on the cell surface as indicated by the red arrow. No QD uptake was observed in the cytosol of the cardiac myocyte. Scale bar = 20 μm.

**Figure 6:**
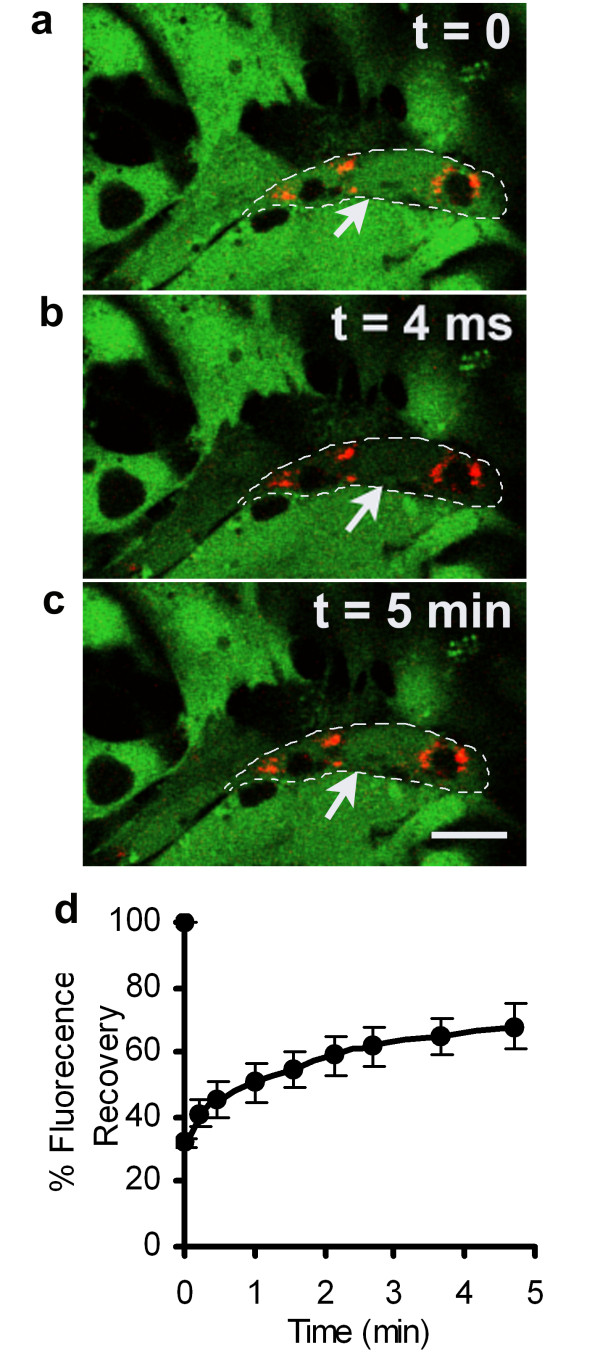
**Functional gap junction mediated MSC- cardiac myocyte communication in QD labeled MSC**. Fluorescence recovery in calcein labeled co-culture with QD labeled MSC (noted by dashed white border and arrow) adjacent to myocytes, **a**. before photobleach **b**. immediately after photobleach and **c**. 5 min. after photobleach. **d**. Corresponding graph illustrating average fluorescence recovery time (n = 6). Scale bar = 20 μm.

Identification of QD labeled MSCs in co-culture with cardiac myocytes was evaluated with fluorescence confocal microscopy, imaging through the Z axis. QD labeled MSCs were identified by punctate red fluorescent cellular inclusions as shown in Figure 4. The QDs appear to be localized to the MSCs. In preliminary studies, using the manufacture's protocol for labeling tumorigenic cell lines, we were unable to intracellularly label the cardiac myocytes with QDs. Figure 5 illustrates the 3D distribution and location of QDs in cardiac myocytes and MSCs 24 hrs post labeling. This figure clearly shows the QD aggregates on the surface of the cardiac myocyte, while the QDs are more diffusely located in the MSC cytosol. Gap junction-mediated cell-to-cell communication between the cardiac myocytes and MSCs was evaluated in the co-culture model using confocal microscopy and a fluorescent dye diffusion assay (fluorescence recovery after photobleaching, FRAP). A representative image of a 7 day co-culture is shown in Figure 6a–c, illustrating the FRAP protocol and fluorescence recovery in the MSC. The graph in Figure 6d shows the average fluorescence recovery over 5 minutes measured in QD labeled MSCs adjacent to cardiac myocytes (n = 6). Fluorescence recovery time is comparable to published results from similar stem cell-myocyte co-culture models [21].

## Discussion

Stem cell transplantation is currently being investigated as a potential therapy for chronic heart failure. While this novel, innovative approach for treating injured or damaged heart muscle has reported positive results with MSCs, the mechanisms underlying functional improvement are not known. This research was initiated as *in vitro *and *in vivo *studies necessary to elucidate stem cell engraftment and function have been limited due to current stem cell labeling and tracking techniques.

Commercially available CdSe/ZnS QDs were used at concentrations of 5 nmol/L (LC) and 20 nmol/L (HC) to evaluate the cytotoxic effects on rat mesenchymal stem cells (MSCs). The two concentrations were selected as they represented one-half and twice the manufacturer recommended QD labeling dose. Adhering to the manufacturer's instructions for QD labeling time of 1 hr we found that QD exposure resulted in a high yield of viable, labeled MSCs that were bright, photo-stable and visible in live cell cultures for up to 7 days. Preliminary studies in our laboratory suggest that alternative cell tracking probes for longer term cell tracking, i.e. 24 hrs, are less photostable with low fluorescence emission intensities when evaluated in 7 day live cell cultures (data not shown). Cytotoxic effects were minimal; QD exposure did not interfere with metabolic activity or significantly affect DNA structure. However, at the higher QD concentration we did find a dose-dependent increase in apoptotic cell death and increase in cytokine release. Similar to recent findings by others [18,20], confocal microscopy and TEM showed that QD aggregates localized in endosomal vesicles in the peri-nuclear region of the MSCs.

At both the low and high concentrations QDs appear to be cytocompatible with the MSCs and capable of labeling proliferating stem cells *in vitro*. These results suggest that when using QDs to label and track stem cells, QD concentration and exposure time should be optimized to reduce cytotoxic effects. The Qtracker cell labeling kit combined QDs with a custom targeting peptide to improve QD solubility and intracellular delivery. With this delivery system QDs had the tendency to aggregate and intracellular QD aggregates were more abundant and appeared larger at higher QD concentrations. This increase in intracellular QD aggregate size and number may have contributed to the observed dose effects. It is possible that lower QD concentrations and longer exposure times may yield smaller QD aggregates and reduced cytotoxic effects with similar QD labeling yield. The development of cell-penetrating QDs may require lower QD labeling concentrations [22], while factors such as surface charge, core size and incubation media have been identified as important for uniform and complete labeling [18,20]. In addition, reports suggest that QDs are sensitive to environmental factors such as pH, salts, oxidation and temperature [23,24]. These factors were not evaluated but should be considered when used with MSCs for *in vitro *and *in vivo *applications.

Our results suggest that labeled MSCs should be used within the first 24 hrs after QD labeling when evaluated in a co-culture system, as detection of QD labeled MSCs decreased as cells proliferated in culture. Documentation by the manufacturer stated that QDs are inherited by daughter cells for at least 6 generations. It is possible that flow cytometry was not sensitive enough to detect intracellular QDs in MSCs as they proliferated over time. It is hypothesized that asymmetric cell division and unequal division of endosomes to daughter cells could result in a dilution of QD labeling as MSCs proliferate [18]. Our results support this as confocal image analysis showed that the number of QD aggregates did not change substantially 72 hrs after labeling and fewer QD labeled MSCs were detected. Yet, it is important to note that for *in vitro *and *in vivo *cell tracking studies, QD labeled MSCs are expected to be transplanted within 24 hrs of QD labeling and to engraft and differentiate in the host environment, maintaining their cellular label.

Results of this study address only QD effects on proliferating MSCs, cell tracking and engraftment in *in vitro *co-cultures. While QDs appear to be safe to use in MSCs, it is believed that a low percentage of transplanted MSCs engraft during cellular cardiomyoplasty. Presently, the mechanism of metabolism or clearance of QDs from transplanted cells *in vivo *is not understood. *In vitro *studies in our laboratory suggest that when compared to MSCs and under similar labeling conditions, cardiac myocytes do not readily endocytose QDs. However, animal studies show that QDs accumulate in bone marrow, spleen and liver for up to 4 months [25]. The outer shell of the QD is inert, while the inner cadmium core is toxic. While it is unlikely that chemical or enzymatic degradation of the outer shell occurs in organs that accumulate QDs, this information is not available. In addition, while increased cytokine release was not significant for LC MSCs both MCP-1 and IL-6 were elevated after HC QD labeling. While increased cytokines did not affect MSC proliferation, this finding may be relevant in applications where QD labeled MSCs are transplanted into injured or diseased tissue where cytokine levels are elevated, contributing to an inflammatory or immune response. Further *in vivo *animal testing is necessary to evaluate QD labeled MSC engraftment and efficacy in damaged heart muscle.

## Conclusion

Results of this study provide new information concerning the cytocompatibility of QDs on MSCs and their use as a label to track MSCs to evaluate MSC function in an *in vitro *cardiac myocyte co-culture. To the author's knowledge, this is the first report showing functional integration of QD labeled MSCs in a cardiac microenvironment. Quantum dot labeled MSCs were bright, photostable and easy to track in live co-cultures providing the opportunity for functional studies in heterogeneous cell cultures. Dose-dependent cytotoxic effects suggest that initial QD exposure be optimized and limited to low concentrations. Future applications of QDs, in addition to long term *in vivo *cell tracking and imaging, may involve combination with drug delivery systems to treat and monitor injured heart tissue.

## Methods

### Cell cultures

MSCs were isolated from 6 week old male Fisher rats using Caplan's method [26] in accordance with the accepted guidelines of the care and treatment of experimental animals at East Carolina University and the National Institutes of Health. Under sterile conditions the femur and tibia were flushed with Dulbecco's Minimal Essential Medium (DMEM, Gibco, Grand Island, NY) supplemented with 10% Fetal Bovine Serum (FBS, Hyclone, Logan, UT) and 1% penicillin, streptomycin and incubated at 37°C, 5% CO_2_. Non-adherent cells were removed at 24 hrs and every 2 days there after for 1 week. Adherent cells were trypsinized, replated for expansion and grown to 80% confluence.

### Quantum dot labeling

MSCs were labeled with Q-Tracker 605 Cell Labeling kit (Invitrogen, Carlsbad, CA). These QDs, approximately 10–15 nm in diameter, are composed of a cadmium selenium core and an inner zinc sulfide shell (CdSe/ZnS). A custom peptide bonded to the QD's outer shell allows the QD to be endocytosed into the cell interior and exist in periplasmic vesicles [9,27]. Growth medium containing 0 nmol/L(Control), 5 nmol/liter (LC) or 20 nmol/L (HC) QDs was added to 1 × 10^6 ^MSCs in suspension and incubated for 1 hr at 37°C, 5% CO_2 _according to the instructions of the manufacturer. The QD concentrations evaluated were one-half (LC) and twice (HC) the manufacturer's recommended labeling concentrations. MSCs were washed, resuspended in full growth media, plated and allowed to expand for 24, 72, and 120 hrs. Observations of live cells were terminated at 120 hrs.

### MSC survival and QD yield

To assess QD yield, retention and MSC viability an Annexin-V-Fluos staining kit (Roche, Mannheim, Germany) and flow cytometry were used at 24, 72 and 120 hrs post QD labeling (n= 3 cell isolations). Flow cytometry identified MSC populations as QD positive or negative and further separated the cells into annexin positive or negative groups. The annexin assay identified MSCs undergoing apoptosis. Briefly, MSCs exposed to media or QDs were trypsinized, counted and washed with PBS (Phosphate Buffered Solution). According to manufacturer's directions, Annexin-V-Fluos labeling solution was added to 2 × 10^5 ^cells in the Control, LC, and HC groups and MSCs were analyzed on a Becton Dickinson FACScan flow cytometer with CellQuest software (BD Biosciences, San Jose, CA).

### Intracellular distribution of QDs

Confocal fluorescence images were acquired with a Zeiss LSM 510 inverted microscope (Zeiss LSM 510, Carl Zeiss, Oberkochen, Germany) equipped with a with a 63X/1.4 NA water immersion objective. Control, LC and HC MSCs were plated on coverslips coated with poly-L-lysine in full growth media and incubated at 37°C, 5% CO_2_. After 24, 72 and 120 hrs the media was removed and cells were rinsed with PBS. Images of QD intracellular distribution in live MSCs at 24 and 120 hrs were acquired for each MSC isolation (n = 3). To observe MSCs under fluorescence microscopy, the MSCs were labeled with 1 μmol/L calcein acetoxymethylester (calcein AM; Invitrogen, Eugene, OR) and imaged with a 488 nm argon excitation laser excitation and 515 ± 15 nm band pass filter. Quantum dots were imaged with a 458 nm argon excitation laser and 580 nm long-pass filter. For each time point and QD concentration, an average of 100 cells were imaged and evaluated to quantify QD aggregates. ImageJ software  was used to evaluate QD location, aggregate number and distribution in MSCs.

Transmission electron microscopy (TEM) was performed to further determine QD location in the MSCs. Twenty four hrs post QD labeling, MSCs were trypsinized, pelleted, washed with PBS, and fixed with 2%glutaraldehyde. Pelleted cells were washed in Na Cacodylate buffer, treated with 1% Osmium tetroxide, rinsed with PBS and dehydrated in graded ethanol. The cell pellet was treated with acetone, and embedded in Spurr's resin. Thin sections (80 nM) were cut and mounted on copper grids. Images were collected at 15,000× to 250,000× on a 60,000 Kv Jeol 1200EX (Jeol Ltd, Waterford, VA) and analyzed with iTEM (Soft Imaging System, Lakewood, CO).

### DNA damage

To assess single and double strand DNA damage a single cell gel electrophoresis assay was used at 72 and 120 hrs post QD labeling (Comet assay kit, Trevigen, Gaithersburg, MD). Per manufacturer's instructions, MSCs exposed to media or QDs were harvested and 100,000 cells per group were pelleted and resuspended in ice cold PBS. As a positive control, a group of Control MSCs were treated with 100 μmol/L hydrogen peroxide (H_2_O_2_, a known DNA oxidizer) for 10 minutes at 4°C, and then washed with PBS. MSCs were plated on pre-treated comet slides, placed in lysis solution for 1 hr at 4°C and in alkaline solution for 40 minutes at 21°C. Electrophoresis was performed at 4°C with 30 V for 45 minutes. Cells were dehydrated in 70% ethanol. Total DNA was stained with SYBR Green.

Comet assay slides were imaged with a Zeiss LSM 510 fluorescence microscope equipped with a 20X/0.50 NA objective and 505 nm long-pass filter. The comet tail moment was analyzed with comet scoring software (Northern Eclipse, North Tonawanda, NY). The tail moment was calculated as the product of the tail length and the fraction of signal in the comet tail [28]. Double and single strand DNA damage was identified by increased dispersion patterns of the comet tail. Three replicate experiments were performed.

### Cytokine release

The inflammatory response of the MSCs to the QDs was evaluated with a rat cytokine/chemokine Linco*plex *kit (Linco Research Inc, St.Charles, MO) and Luminex 100 analyzer (Luminex Corp, Austin, TX). Media from MSCs 24 hrs post QD labeling was removed and spun at 1500 rpm for 5 minutes. The supernatant was removed and assayed for MCP-1, IL-6, IL-1β and TNF-α.

### Cell proliferation

Metabolic activity of MSCs at 24, 72 and 120 hrs was measured with a cell proliferation assay (CellTiter 96 Aqueous One Solution, Promega Corporation, Madison, WI). Control and QD exposed MSCs were added in triplicate to a 96 well plate. Plates were incubated for 24, 72, and 120 hrs at 37°C, 5% CO_2_. At each time point, Aqueous One Solution was added to each well according to manufacturer's instructions, and absorbance was read at 490 nm on a Perkin Elmer plate reader (Perkin Elmer, Inc, Wellesley, MA). Absorbance was directly proportional to metabolic activity. Three replicates of each treatment were completed.

### In vitro model

Rat ventricular cells were isolated and co-cultured as previously described [29]. Briefly, neonatal cardiac myocytes were isolated from the hearts of 1 day-old Sprague-Dawley rats in accordance with accepted guidelines for the care and treatment of experimental animals at the East Carolina University Brody School of Medicine and the National Institutes of Health. Neonatal cardiac myocytes were isolated using a Worthington Neonatal Cardiomyocyte Isolation System (Worthington Biochemical Corp., Lakewood, NJ). The cells were plated on laminin-coated cover slides at 1 × 10^6 ^cells per 22-mm cover slide and grown in Richter (Irvine Scientific, Santa Ana, CA) medium supplemented with 10% fetal calf serum. The cell cultures were maintained for 48 hrs before the QD labeled MSCs were added at a ratio of 1/100 and maintained in co-culture up to 7 days.

A fluorescent dye diffusion assay, fluorescence recovery after photobleaching (FRAP) was used with confocal microscopy to evaluate functional cell-to-cell communication via gap junctions in the cardiac cell cultures as previously described [21,29]. The cells in co-culture were intracellularly labeled with the fluoroprobe calcein AM. MSCs were identified through intracellular QD fluorescence (previously described). Using a high intensity setting for the 488 nm argon laser on the Zeiss LSM 510 microscope, calcein was bleached in MSCs adjacent to neonatal cardiomyocytes. The MSCs demonstrated fluorescence recovery after photobleaching as a result of calcein diffusion from neighboring cardiomyocytes into the MSCs. Functional cell coupling was assessed at room temperature (21°C).

### Statistical analysis

The data are presented as mean ± SEM. The statistical significance was determined using a Student's T-test and analysis of variance (ANOVA) where appropriate. A *p *value less than 0.05 was considered statistically significant.

## Abbreviations

HC - High Concentration 

IL-1β - Interleukin-1 

IL-6 - Interleukin-6

LC - Low Concentration

MCP-1 - Monocyte Chemoattractant Protein-1

MSC - Mesenchymal stem cell

QD - quantum dot 

TNF-α - Tumor Necrosis Factor alpha.

## Competing interests

The author(s) declare that they have no competing interests.

## Authors' contributions

BJMB and WEC initiated these studies. BJMB and APK supervised experimental design, reviewed data and provided statistical support. MCC and PRG carried out the experiments, data analysis and statistics. BJMB drafted and finalized the manuscript. All authors read and approved the final manuscript.
